# Mild polyaddition and polyalkylation based on the carbon–carbon bond formation reaction of active methylene†

**DOI:** 10.1039/c9ra08155k

**Published:** 2019-12-06

**Authors:** Caicai Jiao, Lilong Gao, Bing Yu, Hailin Cong, Youqing Shen

**Affiliations:** College of Materials Science and Engineering, Qingdao University Qingdao 266071 China gaolilong@qdu.edu.cn; Institute of Biomedical Materials and Engineering, Qingdao University Qingdao 266071 China hailincong@yahoo.com; College of Chemistry and Chemical Engineering, Qingdao University Qingdao 266071 China; State Key Laboratory of Bio-Fibers and Eco-Textiles, Qingdao University Qingdao 266071 China; Center for Bionanoengineering and Key Laboratory of Biomass Chemical Engineering of Ministry of Education, College of Chemical and Biological Engineering, Zhejiang University Hangzhou 310027 China

## Abstract

The Michael addition and alkylation reaction of active methylene compounds (AMCs) with two active hydrogens had been investigated extensively in organic chemistry, while the polymerization of AMCs had few studies. Herein, we reported active methylene-based polyaddition and polyalkylation catalyzed *via* an organic superbase under ambient conditions. A model polymerization was first conducted between ethylene glycol diacrylate (EGDA) and methyl cyanoacetate (MCA). The molecular weight (*M*_w_) of the model polymer was up to 50 500 g mol^−1^ with a high yield (99%). Eight AMCs were selected and a high-throughput parallel synthesizing instrument (HTPSI) was used to synthesize semi-library polymers of AMCs and EGDA *via* a Michael type polyaddition. The obtained AMC-based polymers had low cell cytotoxicity. Elastomers with cyanogen groups could be prepared using trimethylolpropane triacrylate (TMPTA) as a crosslinker. Furthermore, three dihalogen compounds were explored to polymerize with MCA and malononitrile *via* alkylation reactions. The pendent cyanogen or ester groups of the polymers could be reduced by lithium aluminum hydride. Novel polymer families were constructed based on the polyaddition and polyalkylation of AMCs.

## Abstract 2

Active methylene-based polyaddition and polyalkylation catalyzed *via* an organic superbase were studied in a high-throughput parallel synthesizing instrument.

## Graphical abstract

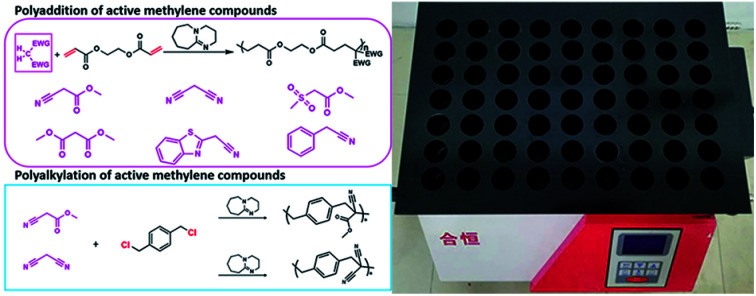

## Introduction

After searching organic chemistry concepts and tools, new polymerization mechanisms and polymers with functional groups were developed. Click polymerization (monomers with two clickable groups),^1,2^ reversible deactivation radical polymerization (RDRP),^3–5^ atom transfer radical polymerization (ATRP)^6,7^ and reversible addition–fragmentation chain transfer polymerization (RAFT)^8–10^ are excellent illustrations.

Among millions of petrochemical products, methylene compounds activated by electron withdrawing groups (EWG) such as acetylacetone, alkyl acetoacetate, dialkyl malonate, alkyl cyanoacetate and malononitrile have active hydrogen on methylene and form carbanions under alkaline conditions. These active methylene compounds (AMCs) have been investigated to synthesize fine chemicals and intermediates by Michael addition,^11–14^ alkylation,^15,16^ Knoevenagel condensation,^17^ aldol condensation,^18^ Dieckmann condensation,^19^ halogenation,^20^ Reformatsky reaction,^21^*etc.* For example, cyanoacrylates, synthesized by cyanoacetates and formaldehyde are widely used adhesives in industrial processing and clinical surgery. The Michael addition reaction of cyanoacetate with acrylonitrile was found to take place by the catalysis of ruthenium complexes or enolatoiron(ii) compounds.^22,23^ In 2009, Narita reported novel fluorinated polymers by the Michael-type anionic polyaddition of 2-trifluoromethylacrylate derivatives with ethyl cyanoacetate (ECA).^23^ However, no study has been reported since then. Polymerization and polymers based on AMCs lack a systematic investigation.

According to previous reports, one methyl acetoacetate could react with two methyl acrylates *via* a Michael addition and two haloalkanes *via* alkylation reactions.^24–27^ Inspired by these studies, we infer that multitudinous AMCs with two active hydrogens will occur *via* a polyaddition (polymerization of AMCs and divinyl compounds) and polyalkylation (polymerization of AMCs with dihaloalkane compounds). Herein, we attempted to construct semi-library polymers based on AMCs.

## Experimental section

### Materials

Methyl cyanoacetate (99%; Macklin; China), malononitrile (99%; Aladdin; China), methyl methanesulfonylacetate (95%; Bidepharm; China), dimethyl malonate (99%; CIVI; China), benzothiazole-2-acetonitrile (98%; CIVI; China), phenylacetonitrile (99%; Jiudingchem; China), ethyl dimethylphosphonoacetate (95%; Energy Chemical; China), fluorene (98%; CIVI; China), benzyl dichloride (98%; Macklin; China), 1,2-bis(2-chloroethoxy)ethane (98%; Energy Chemical; China), 1,4-dibromobutane (98%; Energy Chemical; China), acrylyl chloride (98%; Aladdin; China), lithium aluminium hydride (97%; Energy Chemical; China), trimethylolpropane triacrylate (TMPTA; 99%; AlibabaSJ.com; China), 1,8-diazabicyclo[5.4.0]undec-7-ene (DBU; 98%; CIVI; China) and 4-dimethylaminopyridine (DMAP; 98%; Energy Chemical; China) were used as received. Ethylene glycol (EG; Sinopharm Chemical Reagent; China), triethylamine (TEA; Sinopharm Chemical Reagent; China) and dichloromethane (DCM; Sinopharm Chemical Reagent; China) were used after removing water using a molecular sieve. *N*,*N*-dimethylformamide (DMF) was purchased from Sinopharm Chemical Reagent and used as received. All other reagents were obtained from Shanghai Chemical Reagent and used as received.

### Characterization

^1^H NMR and ^13^C NMR spectra were recorded on a Bruker Avance DMX500 spectrometer in CDCl_3_ or (CD_3_)_2_SO with tetramethylsilane as the internal standard. The molecular weight and molecular weight distribution of these polymers were all determined by gel permeation chromatography (GPC), which was recorded on a Wyatt GPC/SEC-MALS using DMF as the mobile phase with a flow rate of 1.0 mL min^−1^. The solution concentration was 10 mg mL^−1^ and filtered before analysis. The FT-IR spectra were measured by a Nicolet iS 10.

### Synthetic procedures

#### Synthesis of ethylene glycol diacrylate (EGDA)

A total of 5.59 g of dry EG (0.09 mol) was dissolved in 100 mL of anhydrous DCM in a 250 mL round-bottomed flask and then cooled to 0 °C in an ice bath. A total of 38 mL of TEA (0.27 mol) and 24.94 g of acryloyl chloride (0.27 mol) were added to the flask, and the mixture was then stirred for 1 h at 0 °C and 24 h at room temperature. The reaction mixture was filtered to remove triethylamine hydrochloride and was further purified by washing three times with saturated NaHCO_3_. Finally, the organic layer was concentrated and dried at room temperature under vacuum for 1 day. The yield of EGDA was 95%.

##### Model polymerization

Methyl cyanoacetate (MCA, 0.50 g, 5 mmol) and a catalyst (TEA, DMAP and DBU, 0.05 mmol) were dissolved in 20 mL of DCM. Then, 0.85 g of EGDA (5 mmol) was added into each system. A high-throughput parallel synthesizing instrument (HTPSI, Fig. S1†) was used for the reaction at 25 °C. After 48 h, the crude product was washed three times with a brine solution and the organic layer was concentrated and dried at room temperature under vacuum for 1 day. The obtained polymer was named P(MCA-EGDA). The yield of P(MCA-EGDA) was 99%.

##### Reaction kinetics

MCA and DBU were dissolved in chloroform-d followed by the addition of EGDA. At intervals of 1, 2, 3, 4, 6, 12, 24 and 48 h, 0.5 mL of the solution was taken and the reaction was stopped by the addition of acetic acid. The sample was then tested by ^1^H NMR spectrum.

##### Degradation studies of P(MCA-EGDA)

Briefly, 0.01 g of P(MCA-EGDA) was added to a 2 mL centrifuge tube and 20 groups were set under the same conditions. To each centrifuge tube, 100 μL of a 10 mM, pH = 7.4 phosphate buffered saline (PBS) solution was added. The centrifuge tube was placed in a constant temperature shaker at 37 °C, 100 rpm. The PBS solution was changed every 24 h. Masses of dry P(MCA-EGDA) were measured at a predefined time. The degradation fractions were calculated as follows:Degradation fraction (%) = (*M*_0_ − *M*_t_)/M_0_ × 100where *M*_0_ is the mass of the original P(MCA-EGDA) and *M*_t_ is the mass of the undegraded dry P(MCA-EGDA).

### Polyaddition of AMCs with EGDA

The polymerization of 8 AMCs (methyl cyanoacetate, malononitrile, methyl methanesulfonylacetate, dimethyl malonate, benzothiazole-2-acetonitrile, phenylacetonitrile, ethyl dimethylphosphonoacetate and fluorene) with EGDA was conducted in the HTPSI for the parallel polymerization in DCM at 25 °C. The molar ratio of the raw materials was 1 : 1, and DBU (1 mol%) was used as the superbase catalyst. After 48 h, by analyzing the ^1^H NMR spectra, we found that methyl cyanoacetate, malononitrile, methyl methanesulfonylacetate, dimethyl malonate and benzothiazole-2-acetonitrile could conduct a good polymerization. The corresponding polymers were named P(MCA-EGDA), P(MN-EGDA), P(MMSA-EGDA), P(DMM-EGDA) and P(BTAN-EGDA). We inferred that the temperature may have had a great effect on the Michael addition. Then, phenylacetonitrile, ethyl dimethylphosphonoacetate and fluorene with EGDA were used in the HTPSI for the second parallel polymerization in DMF at 70 °C. Only phenylacetonitrile conducted a good polymerization and the obtained polymer was named P(PAN-EGDA). The yields of the six polymers all exceeded 90%.

### Polyalkylation of dihalogens with MCA and malononitrile

The polyalkylation of the dihalogens (benzyl dichloride, 1,2-bis(2-chloroethoxy)ethane and 1,4-dibromobutane) with MCA and malononitrile were conducted in the HTPSI for the parallel polymerization in DCM at 25 °C. The molar ratio of the raw materials was 1 : 1, and DBU (2.1 mol%) was used as the superbase catalyst. After 48 h, by analyzing the ^1^H NMR spectra, we found that only benzyl dichloride conducted a good polymerization with MCA and malononitrile. The corresponding polymers were named P(MCA-BDC) and P(MN-BDC). We inferred that the temperature may have a great effect on the reaction. Then, 1,2-bis(2-chloroethoxy)ethane and 1,4-dibromobutane with MCA and malononitrile were conducted in the HTPSI for the second parallel polymerization in DMF at 70 °C. No polymerization reactions occurred by analyzing the ^1^H NMR spectra. The yields of the two polymers all exceeded 90%.

### Reduction reaction of P(MCA-BDC)

Lithium aluminium hydride (0.78 g, 20 mmol) was dissolved in 30 mL of ether in a 150 mL round-bottomed flask, and the reaction was cooled to 0 °C in an ice bath. P(MCA-BDC) (1.01 g, 5 mmol) was dissolved in 30 mL of anhydrous DCM and the solution was added dropwise to the flask. The reaction was stirred for 0.5 h at 0 °C and 2 h at room temperature, followed by refluxing for 6 h at 60 °C. Finally, the reaction was gradually cooled to room temperature and then quenched by adding water. The reaction mixture was filtered and was further concentrated. Finally, the product was dried at room temperature under vacuum for 1 day. The product was named R-P(MCA-BDC). The yield of R-P(MCA-BDC) was 60%.

### Elastomer preparation

Briefly, 0.12 g of MCA (1.15 mmol) and 1.6 mg of DBU (0.01 mmol) were mixed, followed by the addition of the mixture of 0.03 g of TMPTA (0.1 mmol) and 0.17 g of EGDA (1 mmol). The solution was poured into a mold after thoroughly mixing. Two minutes later, the elastomer was removed from the mold. The yield of the elastomer was 100%.

### *In Vitro* cell viability

Human cervical carcinoma cells (HeLa) were maintained in DMEM medium supplemented with 10% fetal bovine serum (FBS), 100 U per mL penicillin, and 100 mg mL^−1^ streptomycin in a 5% CO_2_ atmosphere at 37 °C. The HeLa cells adherently grew in the cell culture flask, and the cells did not proceed to the next step until the bottom of the bottle was almost completely filled. The *in vitro* cell viability of the polymers against the HeLa cells was determined with the 3-[4,5-dimethylthiazol-2-yl]-2,5-diphenyltetrazolium bromide (MTT) assay. The polymers were cultured in the culture medium for 24 h to obtain their extracts with a concentration of 0, 0.16, 0.31, 0.63, 1.25 and 2.5 mg mL^−1^, respectively. The experiments were started after the cells in the cell culture flask reached 1 million. The 1 million cells were diluted to 10 mL with the culture medium and uniformly filled into 96-well plates at a density of 10 000 cells per well in 100 μL of culture medium. After 24 h of incubation, the culture medium was removed and 100 μL of extracts at various concentrations were added to the well as the experimental group while the pure culture solution and pure sterile water was the control group and blank group, respectively. Approximately 24 h later, 5 mg of MTT was weighed in the dark, 1 mL of PBS and 9 mL of cell culture medium were added, and the mixture was shaken evenly to prepare the solution. Then, the liquid in the 96-well plates was removed and each well was washed three times with PBS. To each well, 100 μL of the MTT solution was added. After a further incubation of 4 h, the flip-back method was used to remove the MTT solution and each well was washed three times with PBS. Then, 100 μL of dimethyl sulfoxide (DMSO) was added to each well and the 96-well plate was placed on a shaker at low speed for 2 min. Finally, the results were measured at 490 nm with a Microplate Spectrophotometer (SpectraMax M3). The cell viability data was calculated according to the following formula:
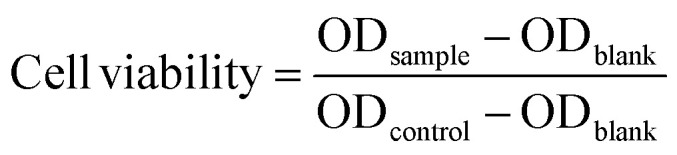
where OD_sample_ is the absorbance of the cells treated with polymer extracts at different concentrations, OD_control_ is the absorbance of the control group and OD_blank_ is the absorbance of the well with no cells, but culture medium.

## Results and discussion

**Fig. 1:**
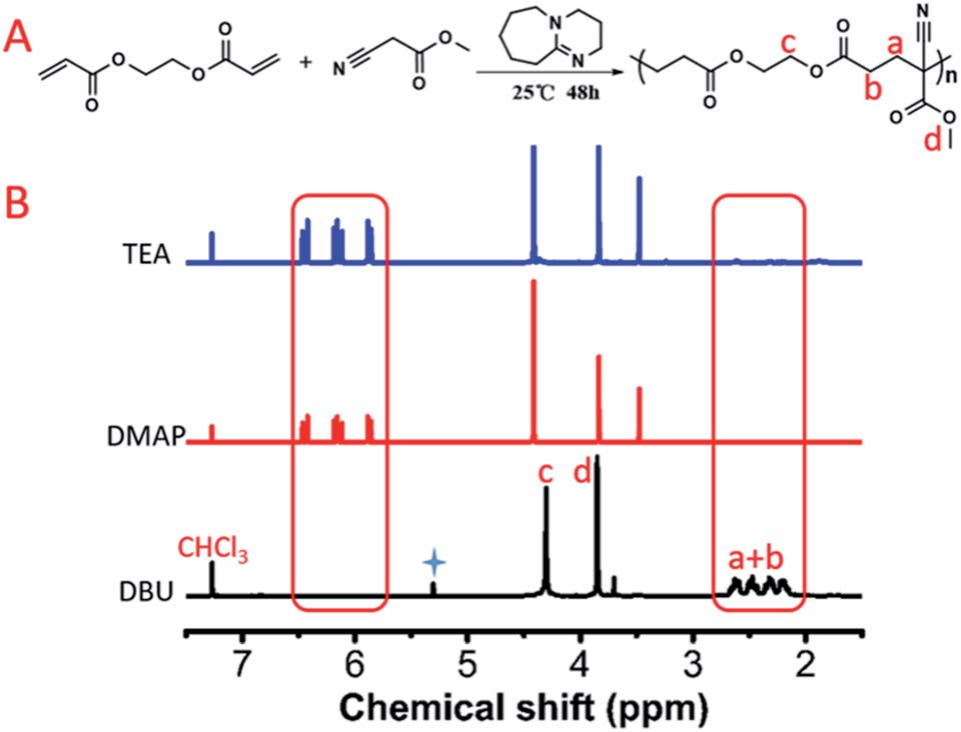
(A) Model polymerization of EGDA and MCA. The obtained polymer was named P(MCA-EGDA). (B) ^1^H NMR spectra of the model reaction catalysed by TEA, DMAP and DBU (* residual solvent).

In this study, we designed a series of polyaddition and polyalkylation reactions for the AMCs. A model polymerization reaction was first conducted to verify the feasibility of an anionic polyaddition between methyl cyanoacetate (MCA) and ethylene glycol diacrylate (EGDA) using an organic base (Fig. 1A). We selected triethylamine (TEA), 4-dimethylaminopyridine (DMAP) and 1,8-diazabicyclo[5.4.0]undec-7-ene (DBU) to screen a suitable organic catalyst. The three catalyst systems were placed in a high-throughput parallel synthesizing instrument (HTPSI, Fig. S1, ESI†) to ensure consistency of the reaction conditions (MCA : EGDA = 1 : 1, solvent: *N*,*N*-dimethylformamide, temperature: 25 °C, catalyst: 1 mol%). As shown in Fig. 1B, for the reaction systems catalysed by TEA and DMAP, the carbon–carbon double bond of EGDA demonstrated almost no reduction and no new carbon–carbon single bonds were formed, which demonstrated that TEA and DMAP could not catalyse the polymerization of MCA and EGDA. Meanwhile, DBU, an organic superbase, catalysed the Michael type polyaddition within 48 hours. Through a model polymerization, we recognized that alkalinity had a great effect on the Michael addition, whereas a higher alkalinity resulted in a higher catalytic activity. Therefore, we selected DBU as the catalyst for the AMCs-based polymerization. In the presence of DBU, a AB type dimer formed due to the addition reaction of the MCA (AA monomer) anion and EGDA (BB monomer). Next, a DBU salt of the MCA moiety was afforded by an ion transfer to form an intermediate. The addition of EGDA with the intermediate yielded a diaddition compound, followed by affording the MCA·DBU salt again. With an increase in time, the relative molecular weight of the polymer and the monomer conversion rate increased gradually. The polymerization mechanism of MCA and EGDA was an anionic polyaddition.

**Fig. 2:**
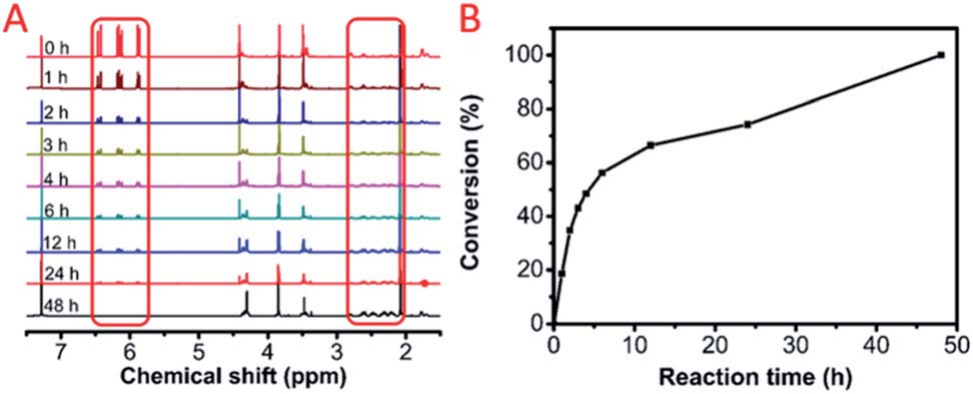
(A) ^1^H NMR spectra of the model polymerization with different times. (B) Conversion of C=C calculated by ^1^H NMR.

**Fig. 3:**
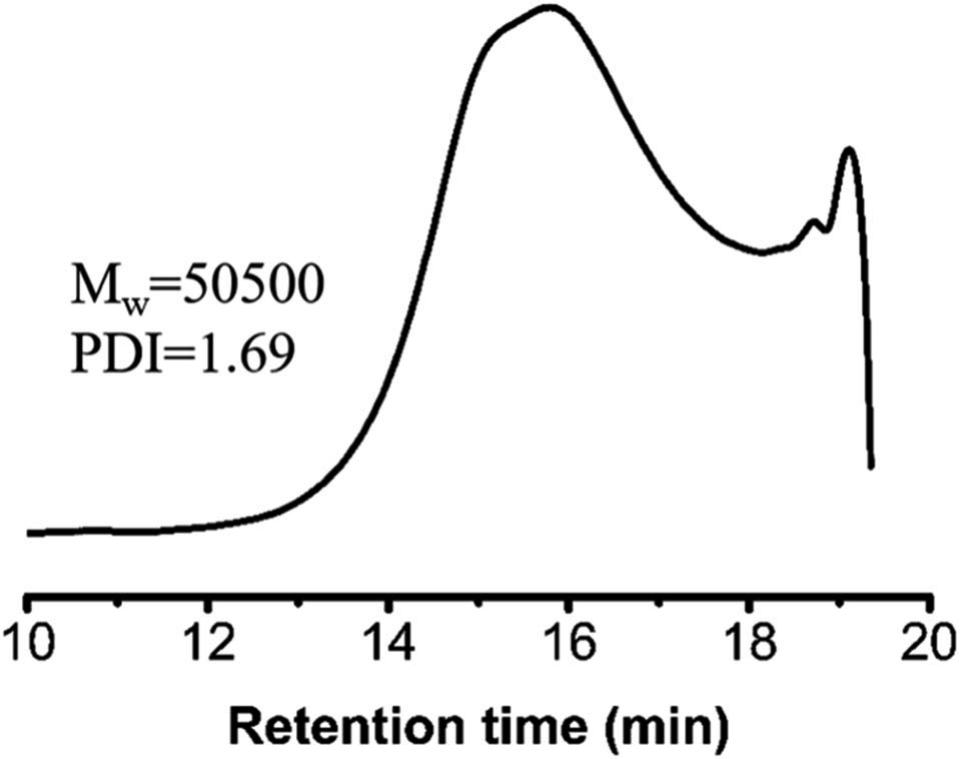
The gel permeation chromatography (GPC) curve of the model polymer, P(MCA-EGDA).

We further investigated the polymerization kinetics of MCA and EGDA (Fig. 2A). The conversion of the carbon–carbon double bond was calculated by ^1^H NMR at different reaction times (Fig. 2B). In the first hour, 19% of the C=C bonds transformed into C–C bonds by the Michael addition. On the first day, 74% of the C=C bonds occurred due to the Michael addition. The final polymerization time was set at 48 hours. The weight-average molecular weight of the obtained polymer catalyst by DBU was up to 50 500 g mol^−1^ with a molecular weight distribution of 1.69 (Fig. 3). After the analysis of the ^1^H NMR (Fig. 1B) and ^13^C NMR (Fig. S3b, ESI†) spectra as well as the unimodal GPC curve (Fig. 3), we believed that it was feasible for polymerization based on the AMCs catalyst by DBU.

Inspired by the above results, we retrieved and selected 7 other compounds (Table 1) to polymerize with EGDA. The HTPSI was utilized to improve the efficiency of the polymerization. The temperature was set at 25 °C. After the 48 hours reaction, we found that malononitrile, methyl methanesulfonylacetate, dimethyl malonate and benzothiazole-2-acetonitrile conducted a good polymerization and the corresponding polymers had a high molecular weight (Table 1). The obtained AMC polymers could be dissolved in solvents commonly used in the laboratory such as *N*,*N*-dimethylformamide, dichloromethane, tetrahydrofuran, acetone, ethanol, *etc.* Unfortunately, no polymerization reactions occurred or only oligomers were synthesized after analyzing the ^1^H NMR spectra and GPC curves of the reaction systems of phenylacetonitrile (PAN), ethyl dimethylphosphonoacetate (DMPA) and fluorene. We inferred that the temperature may have had a great effect on the Michael addition. The HTPSI was set at 70 °C for the second parallel polymerization of PAN, DMPA and fluorene. The polymers based on PAN were detected by GPC (molecular weight more than 23 000 g mol^−1^) and the carbon–carbon double bonds of EGDA disappeared. However, DMPA and fluorene could not generate a polyaddition with EGDA at 25 °C or 70 °C. The ^1^H NMR spectra, ^13^C NMR spectra and GPC curves of the successfully synthesized AMC-based polymers are shown in the ESI.†

**Table 1:** The AMC polyaddition with EGDA^a^

Monomer	Temperature (°C)	*M*_w_ (×10^3^ g mol^−1^)	PDI
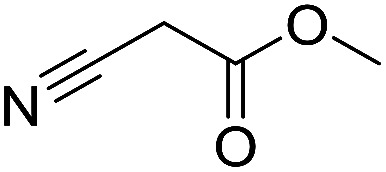	25	50.5	1.69
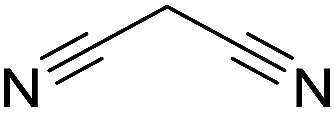	25	30.6	1.67
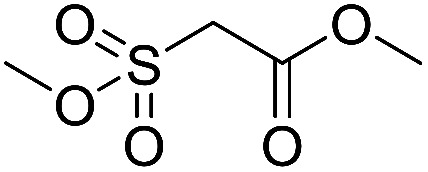	25	31.8	1.45
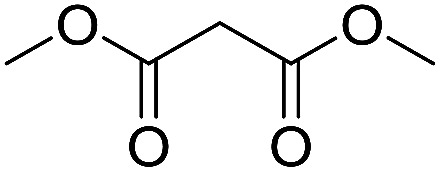	25	37.6	1.52
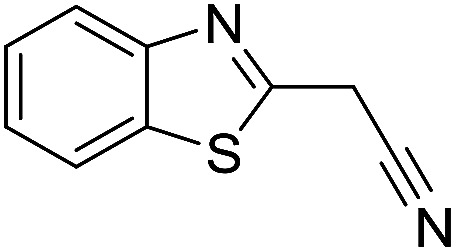	25	34.2	1.57
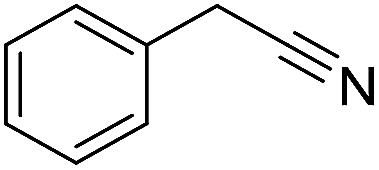	70	23.1	1.33
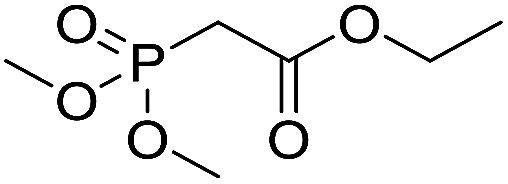	70	N	N
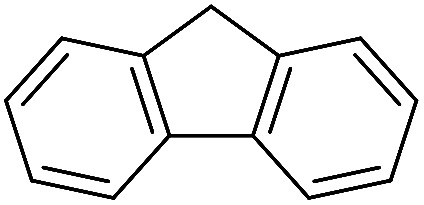	70	N	N

a The molecular weight and PDI were measured by GPC with linear polystyrene as the calibration standard, DMF as the mobile phase, a flow rate of 1.0 mL min^−1^ and a temperature of 50 °C.

Furthermore, we developed an elastomer with cyanogen groups by introducing trimethylolpropane triacrylate (TMPTA) as the crosslinker into the MCA, EGDA and DBU system as shown in Fig. S14.† The elastomer was formed *in situ* after dozens of seconds in an ambient environment and had a high transparency, which may be applied in flexible electronics, optics, bionics, biomedicine, *etc.*

**Fig. 4:**
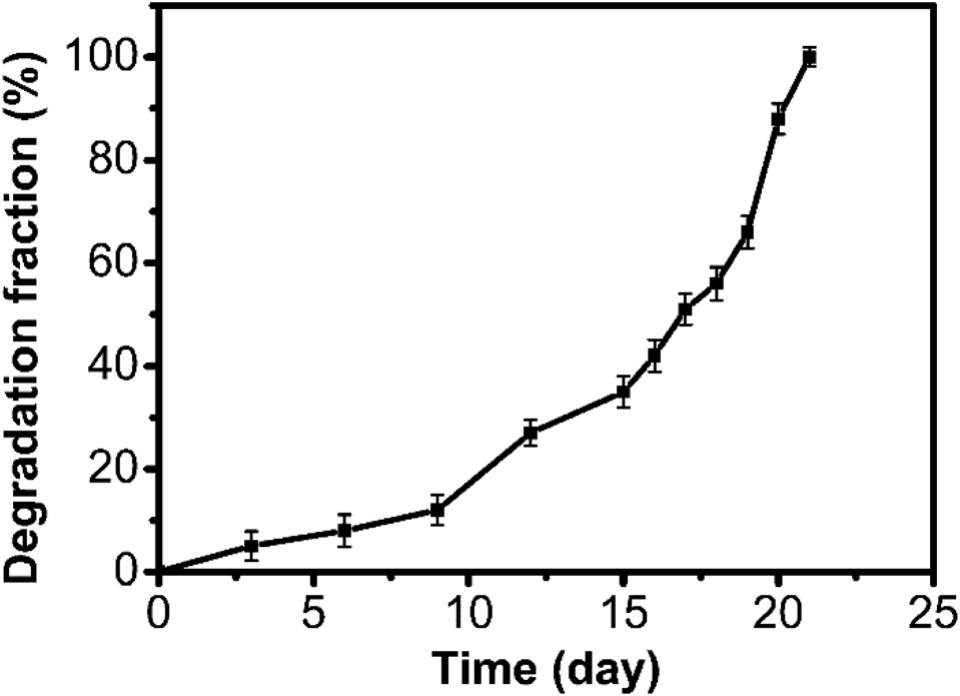
Degradation of P(MCA-EGDA) in phosphate buffered saline (10 mM, pH = 7.4, 37 °C).

Meanwhile, we investigated the degradability of the polyaddition product of P(MCA-EGDA). By immersing the polymer in phosphate buffered saline (10 mM, pH = 7.4, 37 °C), the total degradation time was about 21 days (Fig. 4). The result demonstrated that the AMC-based polymers had a great degradability.

**Fig. 5:**
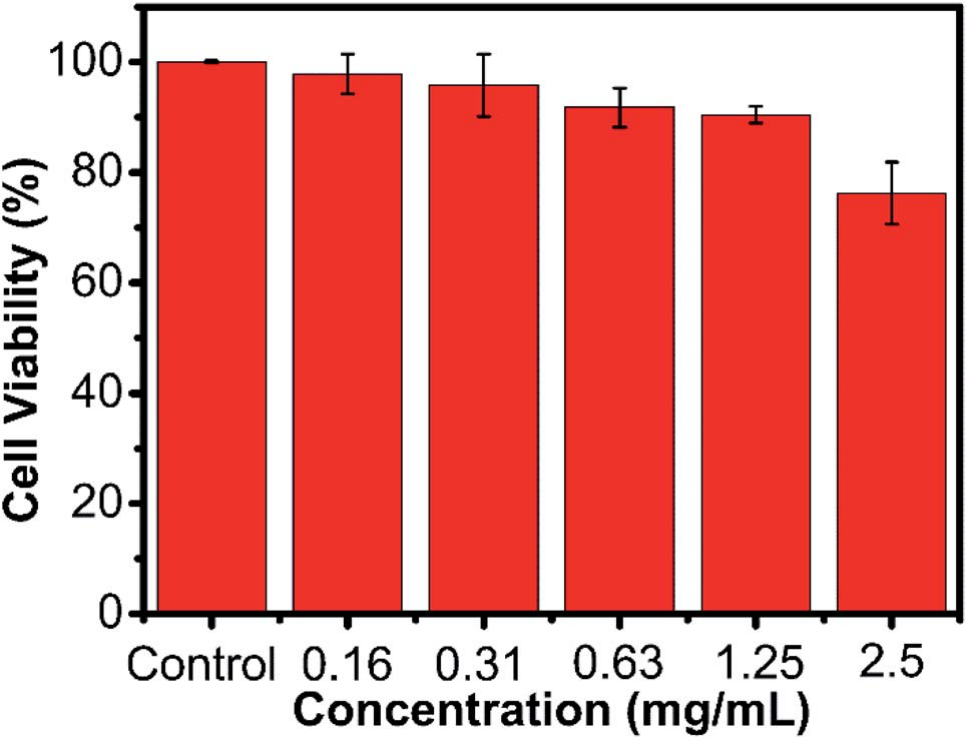
Cell viability of P(MCA-EGDA) against the HeLa cells.

For the potential application of biomedicine, we tested the cell viability of the AMC-based polymers by a MTT assay, which incubated the HeLa cells in various concentrations of the polymer. As shown in Fig. 5 and Fig. S15–S19,† these 6 obtained polymers had little cytotoxicity to the HeLa cells. The low cytotoxicity and degradability demonstrated that the AMC-based polymers had a great potential application as biomaterials.

**Fig. 6:**
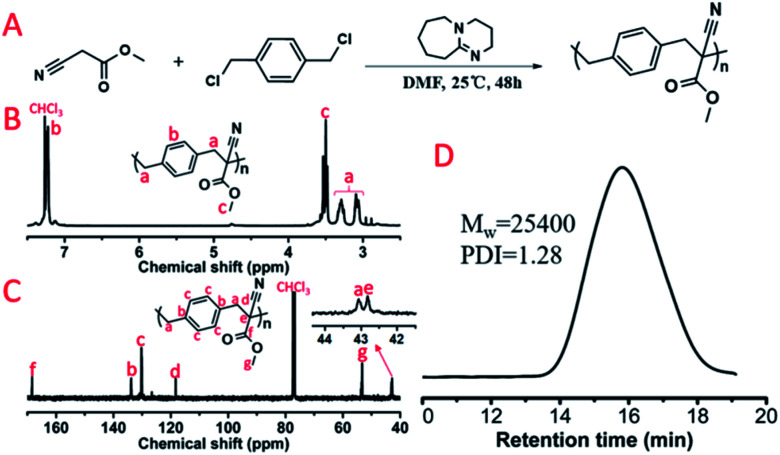
(A) Polyalkylation of MCA with BDC. The obtained polymer was named P(MCA-BDC). (B) ^1^H NMR spectrum and (C) ^13^C NMR spectrum of P(MCA-BDC). (D) GPC curve of P(MCA-BDC).

Subsequently, dihalogen compounds were explored to polymerize with MCA and malononitrile *via* alkylation reactions. Benzyl dichloride (BDC), 1,2-bis(2-chloroethoxy)ethane (BCEE) and 1,4-dibromobutane (DBB) were selected and the polymerization systems were placed in the HTPSI (Table S1†). The molar ratio of DBU was 2.1 equivalents of the dihalogen compounds in order to react with the HCl or HBr byproduct. BCEE and DBB had no polymerization with MCA and malononitrile (25 °C and 70 °C), we obtained 2 new polymers by orthogonal polymerization. In Fig. 6D, the weight-average molecular weight of P(MCA-BDC) was up to 25 400 g mol^−1^ with a molecular weight distribution of 1.28. After the analysis of the ^1^H NMR (Fig. 6B) and ^13^C NMR (Fig. 6C) spectrum as well as the unimodal GPC curve (Fig. 6D), we believed it was feasible for polyalkylation based on the AMC catalyst by DBU. The ^1^H NMR spectrum, ^13^C NMR spectrum and GPC curve of P(MN-BDC) are shown in the ESI.†

**Fig. 7:**
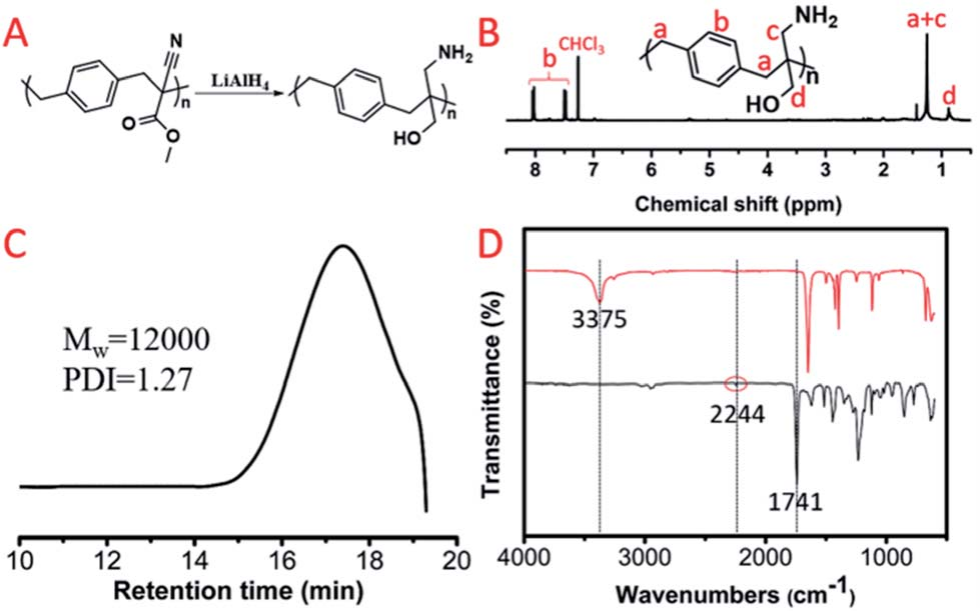
(A) Reduction product of P(MCA-BDC): R-P(MCA-BDC). (B) ^1^H NMR spectrum of R-P(MCA-BDC). (C) GPC curve of R-P(MCA-BDC). (D) FT-IR spectra of P(MCA-BDC) and R-P(MCA-BDC).

For these polyalkylation products, their main chain was composed of a carbon–carbon bond that couldn't be hydrolyzed in water, while the pendent groups as well as the cyanogen and ester groups could be reduced by lithium aluminium hydride. We selected P(MCA-BDC) as a model polymer to conduct the reduction reactions for post-functionalization. The ^1^H NMR spectrum, GPC curve and FT-IR spectrum of the reduction product, R-P(MCA-BDC), are shown in Fig. 7. After the reduction, the proton peak of the ester group (Hc, Fig. 6B) disappeared, indicating that the ester groups were successfully reduced. The molecular weight of P(MCA-BDC) was 25 400 g mol^−1^ (PDI = 1.28) (Fig. 6D) and the molecular weight of R-P(MCA-BDC) was 12 000 g mol^−1^ (PDI = 1.27) after the reduction (Fig. 7C). The FT-IR tests further proved the structure of R-P(MCA-BDC). In Fig. 7D, the absorption of the C=O stretch at 1741 cm^−1^ and the C≡N stretch at 2244 cm^−1^ disappeared after the reduction. Then, the new peaks at 3375 cm^−1^ were attributed to the stretching vibration of OH and NH_2_. These facts indicated that the ester groups were successfully reduced to hydroxyl groups, and the cyanogen groups were successfully reduced to amino groups. The reduction products with multiple OH and NH_2_ groups may have a great application in gene delivery^28,29^ and antibacterial materials.^30^

## Conclusions

In summary, we reported a superbase-catalyzed, AMC-based polyaddition and polyalkylation. After the model polymerization, we selected an organic superbase DBU as an efficient catalyst for the polyaddition and polyalkylation. The optimum polymerization time was 48 h. Therefore, we successfully constructed a semi-library of the AMC-based polyaddition products using HTPSI. The obtained polymers had little cell cytotoxicity and may have an application in biomedicine. An elastomer was formed *in situ* using TMPTA as the crosslinker. In addition, the dihalogen compounds resulted in a polyalkylation with the AMCs. Two novel polymers were synthesized by the polyalkylation. After the reduction reaction, multifunctional derivates with OH and NH_2_ were obtained for the potential biomedical application.

## Conflicts of interest

There are no conflicts to declare.

## Supplementary Material

RA-009-C9RA08155K-s001
