# Supplementary material for: Mild polyaddition and polyalkylation based on the carbon–carbon bond formation reaction of active methylene
Source: RSC Adv. 2019 Dec 6;9(69):40455–61. doi: 10.1039/c9ra08155k (PMC9076256; doi:10.1039/c9ra08155k)
Supplement: RA-009-C9RA08155K-s001 [file RA-009-C9RA08155K-s001.pdf]

Electronic Supplementary Material (ESI) for RSC Advances.
This journal is © The Royal Society of Chemistry 2019

Electronic Supporting Information (ESI)

Mild polyaddition and polyalkylation based on carbon-carbon bond formation reaction of active methylene

Caicai Jiao, Lilong Gao,* Bing Yu, Hailin Cong* and Youqing Shen

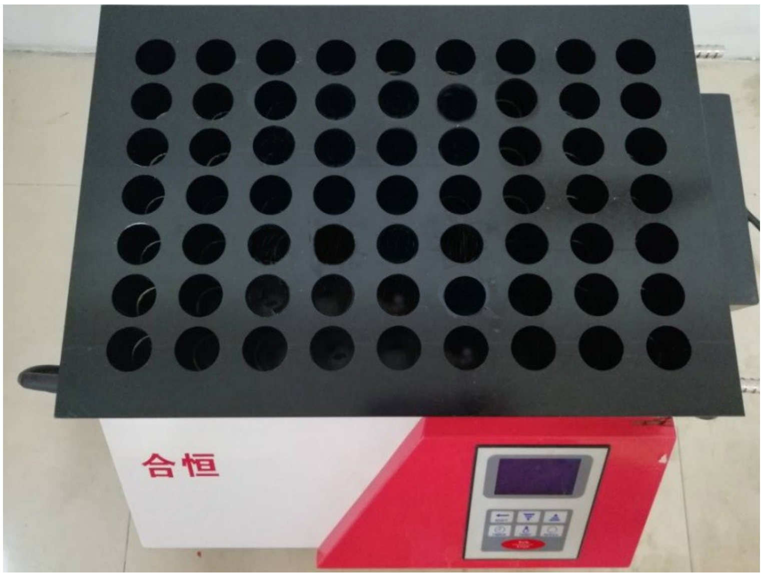

Fig. S1 HTPSI for polymerization of AMCs.

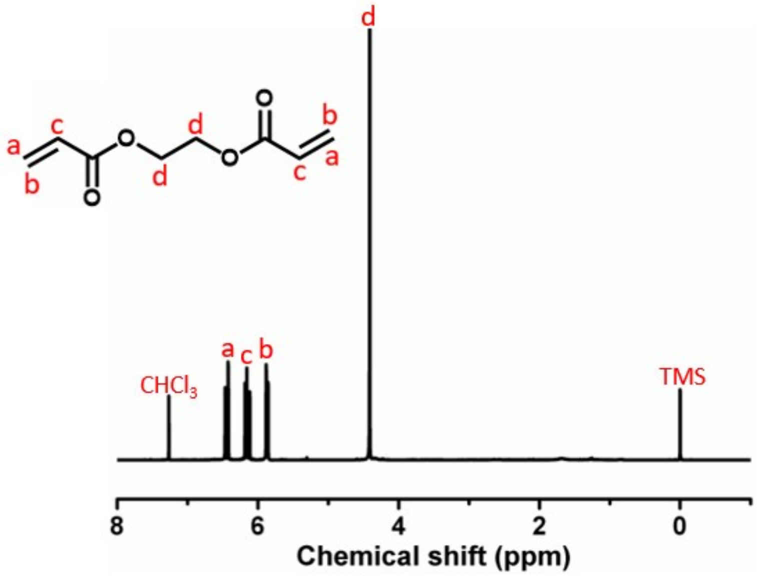

Fig. S2 ^{1}H NMR spectra of EGDA.

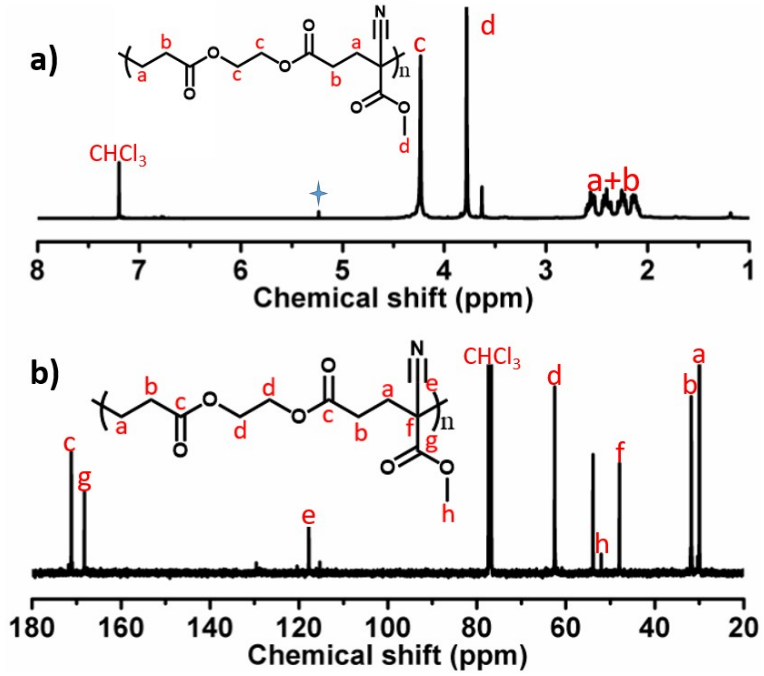

Fig. S3 (a) ^{1}H NMR spectra and (b) ^{13}C NMR spectra of P(MCA-EGDA).

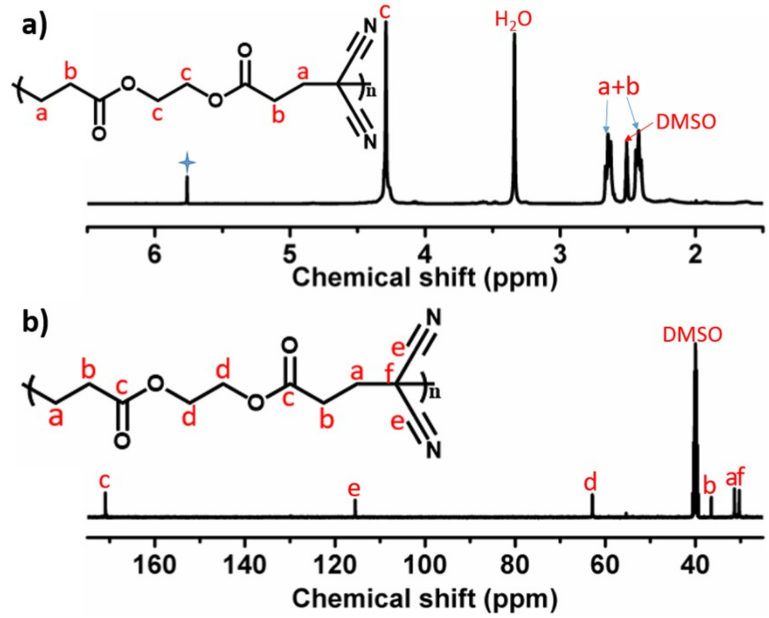

Fig. S4 (a) ^{1}H NMR spectra and (b) ^{13}C NMR spectra of P(MN-EGDA).

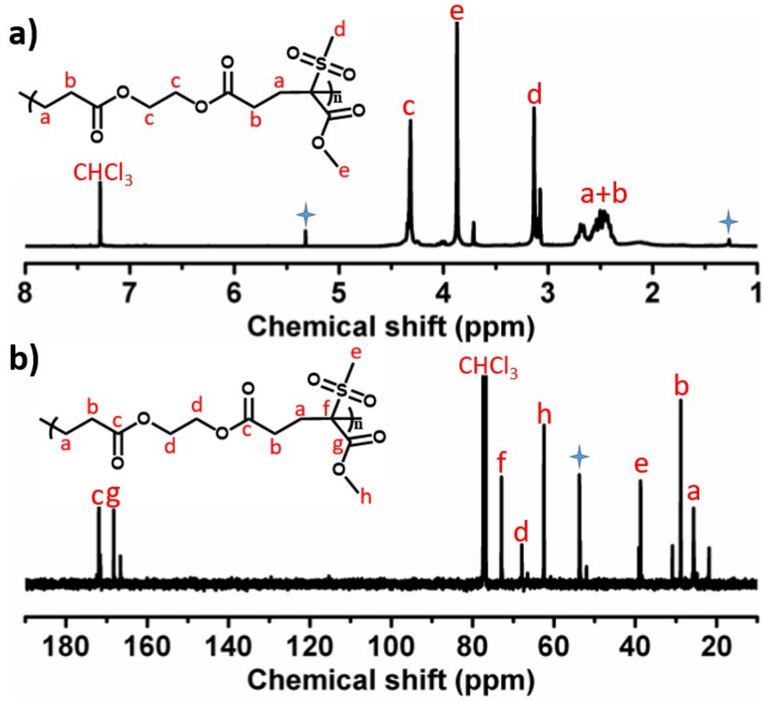

Fig. S5 (a) ^{1}H NMR spectra and (b) ^{13}C NMR spectra of P(MMSA-EGDA).

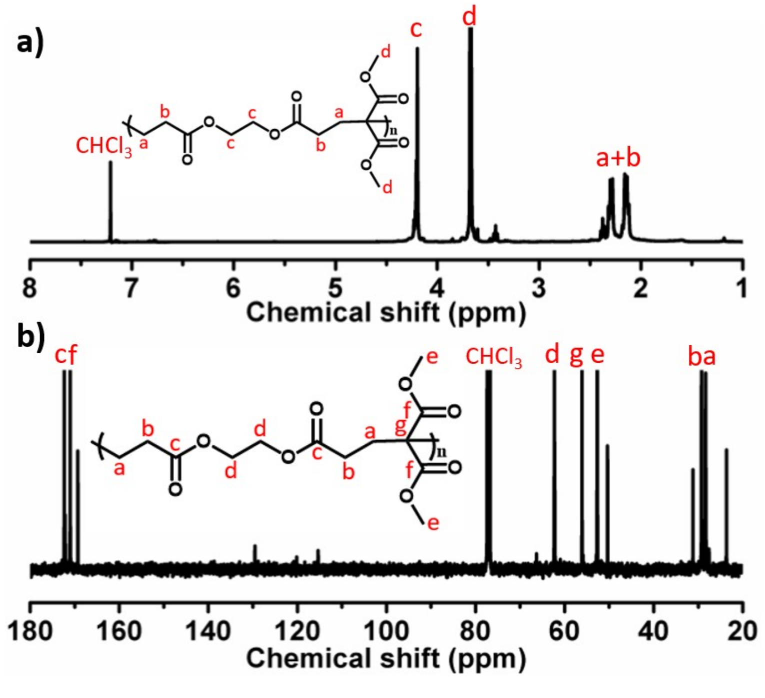

Fig. S6 (a) ^{1}H NMR spectra and (b) ^{13}C NMR spectra of P(DMM-EGDA).

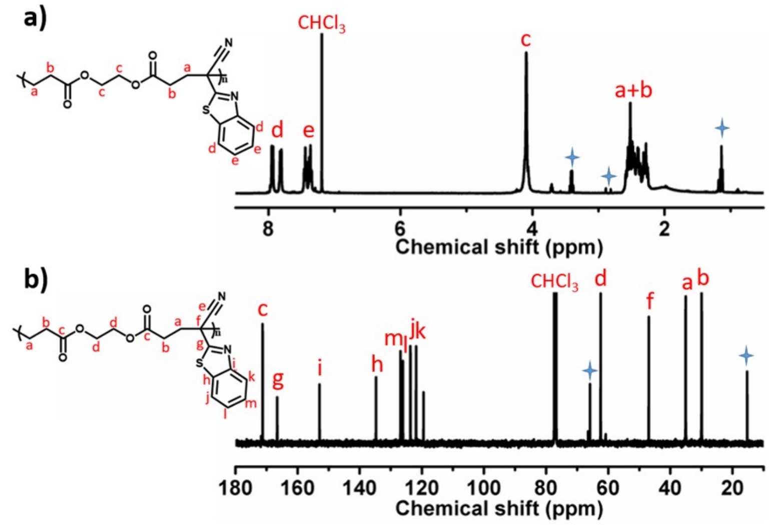

Fig. S7 (a) ^{1}H NMR spectra and (b) ^{13}C NMR spectra of P(BTAN-EGDA).

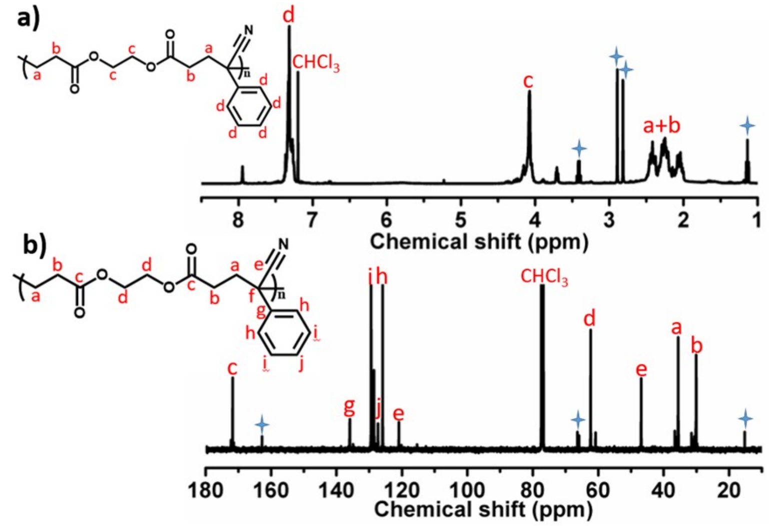

Fig. S8 (a) ^{1}H NMR spectra and (b) ^{13}C NMR spectra of P(PAN-EGDA).

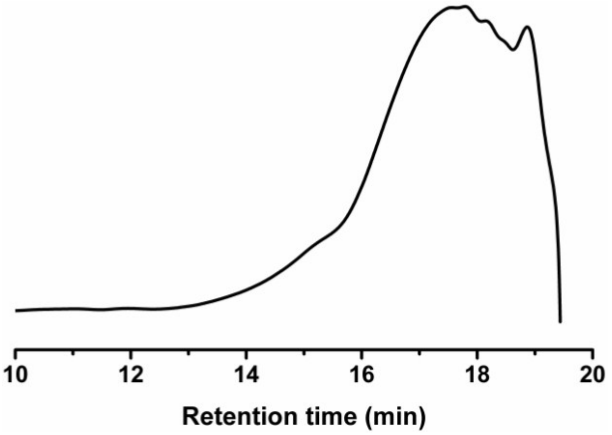

Fig. S9 GPC curve of P(MN-EGDA).

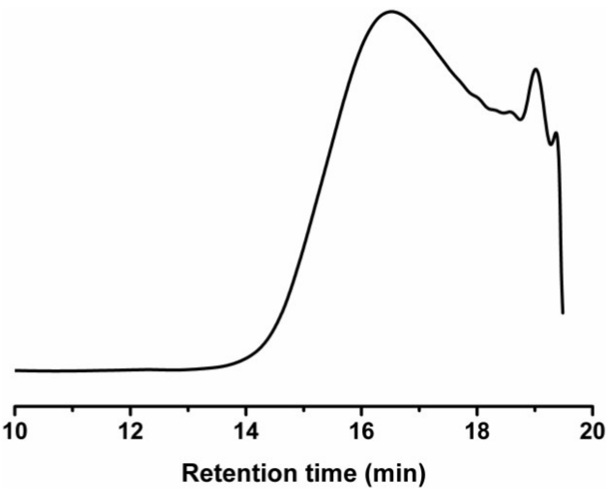

Fig. S10 GPC curve of P(MMSA-EGDA).

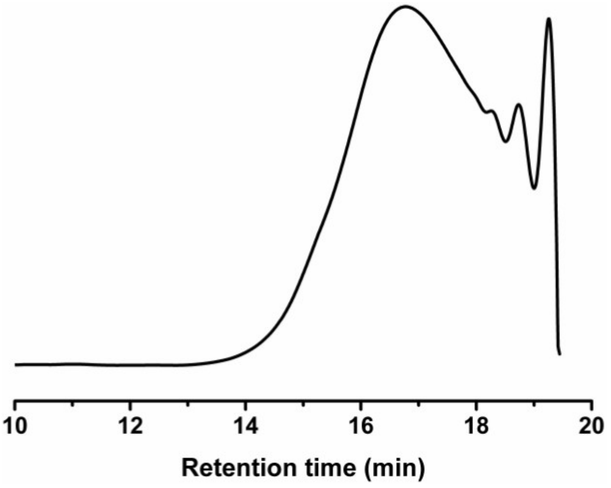

Fig. S11 GPC curve of P(DMM-EGDA).

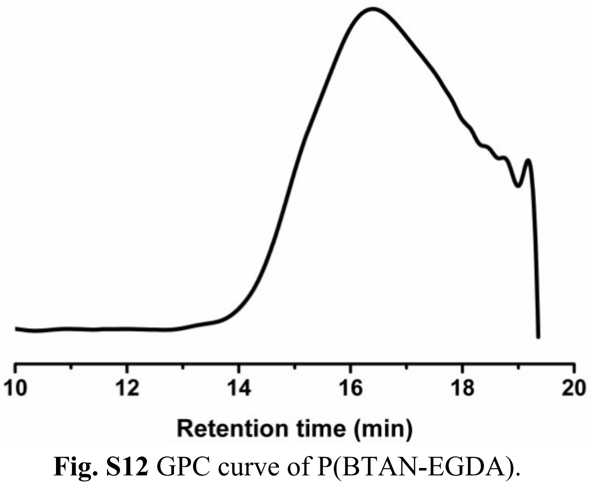

Fig. S12 GPC curve of P(BTAN-EGDA).

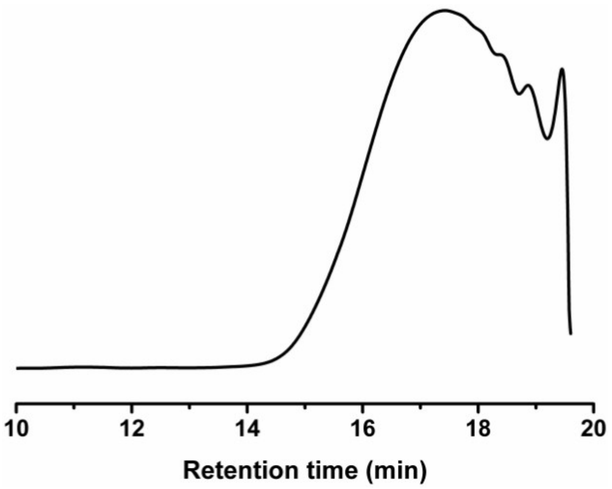

Fig. S13 GPC curve of P(PAN-EGDA).

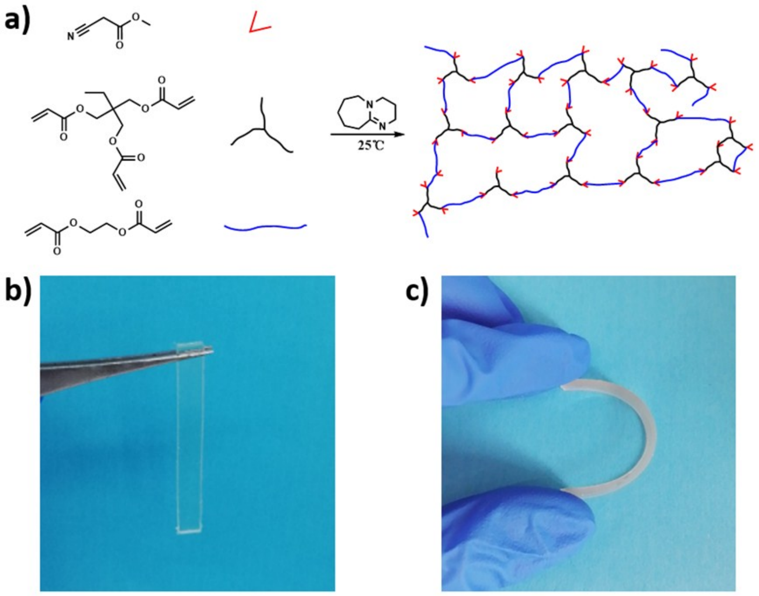

Fig. S14 (a) Synthesis of elastomer with cyanogen groups, MCA: TMPTA: EGDA: DBU=1.15: 0.1: 1: 0.01. (b) Image of elastomer with high transparency and (c) Image of elastomer bending under internal force.

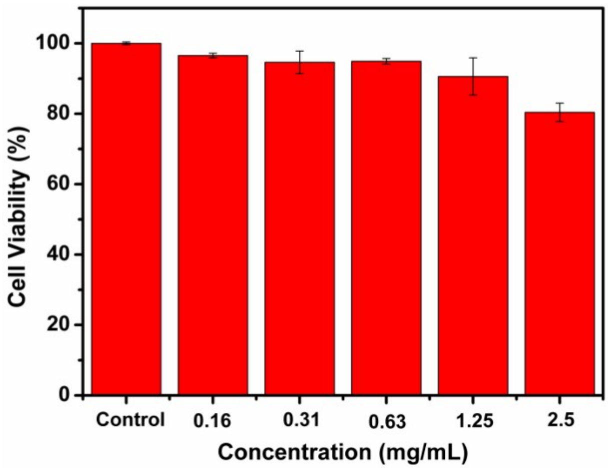

Fig. S15 Cell viability of P(MN-EGDA) against Hela cells.

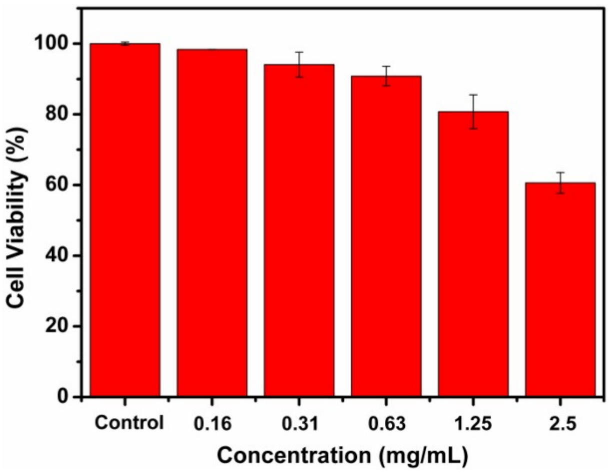

Fig. S16 Cell viability of P(MMSA-EGDA) against Hela cells.

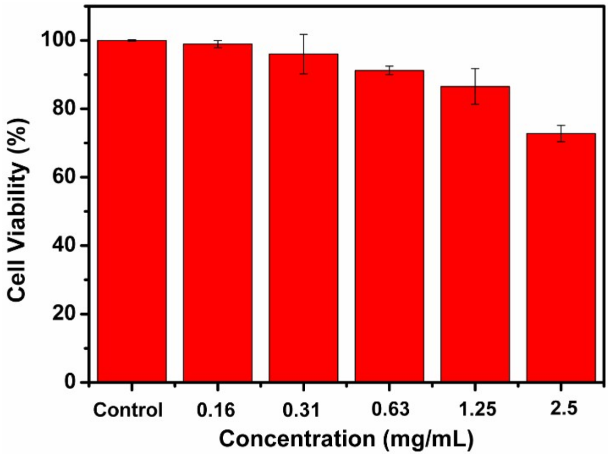

Fig. S17 Cell viability of P(DMM-EGDA) against Hela cells.

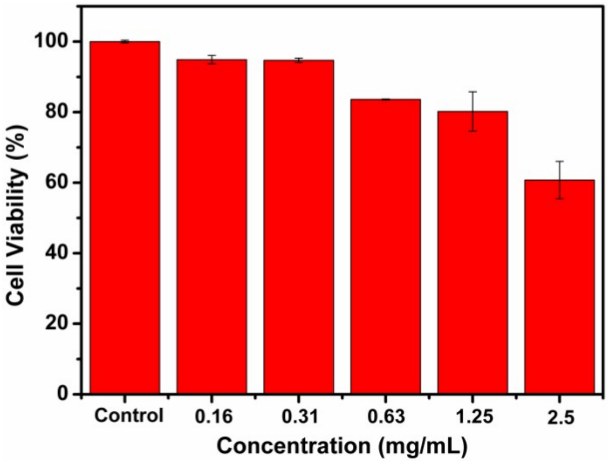

Fig. S18 Cell viability of P(BTAN-EGDA) against Hela cells.

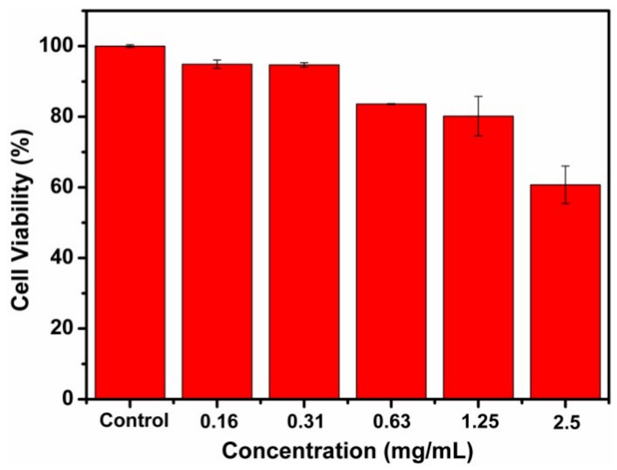

Fig. S19 Cell viability of P(PAN-EGDA) against Hela cells.

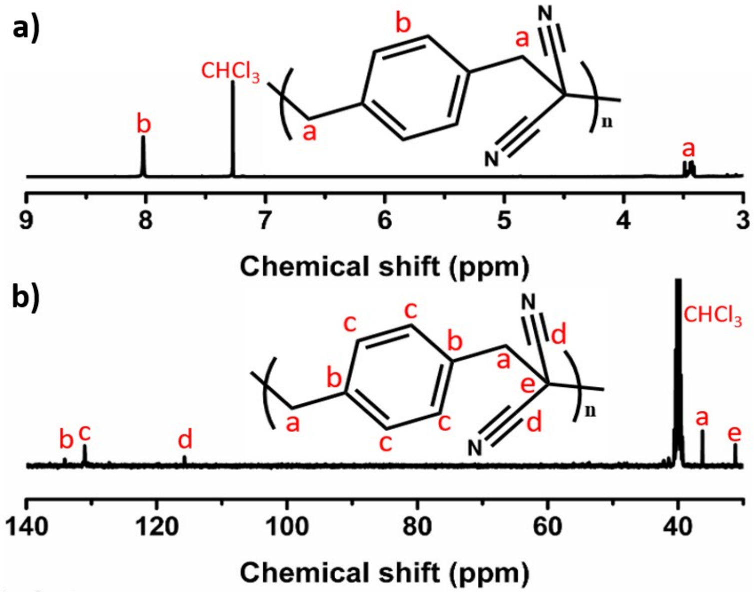

Fig. S20 (a) ^{1}H NMR spectra and (b) ^{13}C NMR spectra of P(MN-BDC).

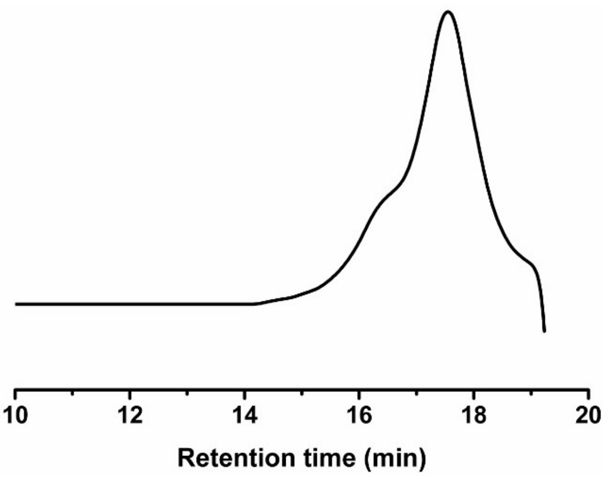

Fig. S21 GPC curve of P(MN-BDC).

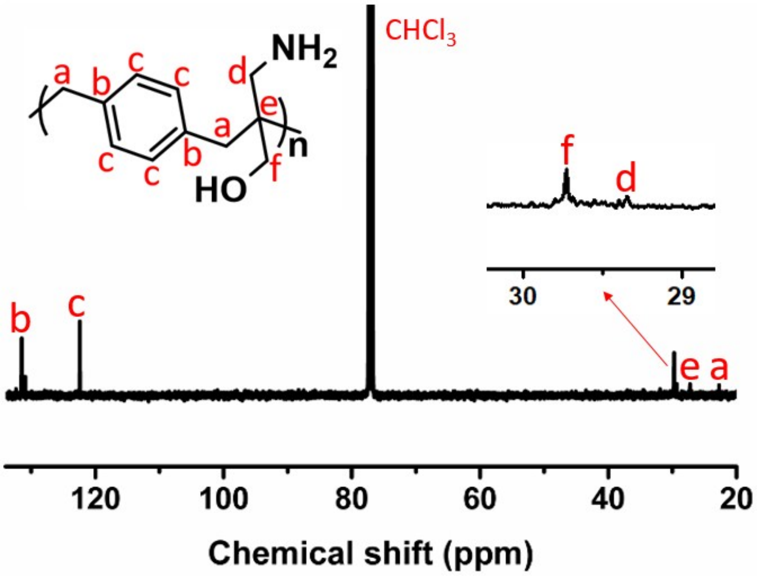

Fig. S22 ^{13}C NMR spectra of R-P(MCA-BDC).

Table S1. The polyalkylation AMCs with dihalogen compounds.

Monomer	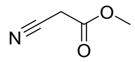		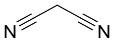	
	M_w (×10^{3}g/mol)	PDI	M_w (×10^{3}g/mol)	PDI
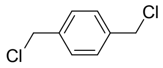	25.4	1.28	12.1	1.22
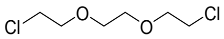	N	N	N	N
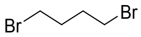	N	N	N	N

Molecular weight and PDI were measured by GPC: linear polystyrene as the calibration standard, DMF as mobile phase, flow rate of 1.0 mL/min, temperature 50 ^{o}C.
